# Comparative analysis of volatile composition and anticholinesterase activity of Egyptian *Hedychium coronarium* and *Alpinia zerumbet* using chemometric assessment of extraction techniques

**DOI:** 10.1038/s41598-026-51750-1

**Published:** 2026-05-15

**Authors:** Esraa A. Shahat, Iriny M. Ayoub, Riham O. Bakr, Haidy A. Gad, Omayma A. Eldahshan, Abdel Nasser B. Singab

**Affiliations:** 1 Pharmacognosy Department, Faculty of Pharmacy, October University for Modern Sciences and Arts (MSA), Giza, Egypt; 2 Pharmacognosy Department, Faculty of Pharmacy, Ain Shams University, Cairo, Egypt; 3 Centre for Drug Discovery Research and Development, Ain Shams University, Cairo, 11566 Egypt

**Keywords:** Essential oil, Hedychium coronarium, Alpinia zerumbet, Acetylcholine esterase inhibitor.\, Antioxidant, GC/MS, Headspace

## Abstract

*Hedychium coronarium* and *Alpinia zerumbet* are rhizomatous plants belonging to the family Zingiberaceae. They are rich in essential oils. Hydrodistillation and headspace (HS) were employed for the extraction of the essential oils from the leaves and rhizomes of both plants. Essential oils were then analysed by GC/MS and the obtained results were subjected to chemometric analysis. The oil samples obtained by hydrodistillation were tested for their antioxidant and anti-cholinesterase activities using oxygen radical antioxidant capacity assay and colorimetric inhibition kit, respectively. Hydro-distilled oil from *H. coronarium* leaves showed the presence of forty-seven compounds (97.73%) compared to sixteen compounds (99.17%) in the HS volatiles. Meanwhile, hydro-distilled oil from *H. coronarium* rhizomes showed the presence of thirty-nine components (95.84%) compared to fifteen compounds (98.68%) in the HS volatiles with 1,8-cineole (41.69% and 58.41%) as the major component in both samples. *A. zerumbet* leaves oil showed the presence of nineteen compounds (94.54%) in the hydrodistilled oil and twenty-one compounds (99.53%) in the HS volatiles. Besides, the rhizomes hydrodistilled essential oil showed twenty-three compounds (91.85%) and sixteen compounds (100%) in the HS with 1,8-cineole (20.78% and 23.63%) representing the major component in both samples. Chemometric analysis of the results provided a clear and statistically robust discrimination between the hydrodistilled and headspace-isolated oil samples demonstrating that extraction method is a primary determinant of volatile profile. Principal component analysis score plot explained 75% of total variance and distinctly separated samples into four main clusters with key discriminating components *β*-pinene, and caryophyllene were the major responsible for the segregation of *H. coronarium* leaves by headspace and hydrodistillation extraction methods, respectively, besides, 1,8-cineole accounted for the discrimination of *H. coronarium* rhizomes extracted by hydrodistillation. Hierarchial cluster analysis (HCA) fully supported this classification, confirming consistent grouping patterns across both analyses. Importantly, *A. zerumbet* oils from both extraction methods clustered closely, indicating compositional stability, whereas *H. coronarium* samples showed strong method-dependent divergence. *A. zerumbet* rhizomes oil showed the strongest activity as anticholinesterase with IC_50_ of 0.54 ± 0.02 µg/mL while *H. coronarium* rhizomes showed the strongest antioxidant activity (10.06 ± 0.16 TE µM/L). *H. coronarium* leaves and *A. zerumbet* rhizomes showed anticholinesterase and antioxidant activities to such an extent that may make them a useful adjuvant in the treatment of cognitive diseases such as Alzheimer’s.

**Supplementary Information:**

The online version contains supplementary material available at 10.1038/s41598-026-51750-1.

## Background

Essential oils are aromatic, volatile liquids that have been utilized for centuries in traditional medicine due to their therapeutic properties. Nowadays essential oils occupy an outstanding position in different industries such as cosmetics, perfumes, and drugs^1^. Furthermore, they act as a main stone in alternative medicine and natural therapies^2^.

In recent years, plant-derived bioactive compounds are increasingly explored as natural antioxidants which improve oxidative stability in food preservation and quality monitoring. For example, extracts from *Peganum harmala* incorporated into sago starch bionanocomposite films enhanced oxidative stability of chicken fillets^3^, while anthocyanins from *Berberis vulgaris* were used in gelatin-based intelligent films for real-time monitoring of veal freshness^4^. Similarly, essential oils rich in volatile bioactive constituents have also attracted attention as promising natural antioxidant sources.

Essential oils can be obtained from plants by different extraction methods, including hydrodistillation, steam distillation, solvent extraction, and headspace solid phase microextraction (HS-SPME)^5^. Previous studies indicated that the chemical profile of the essential oil is greatly affected according to the extraction method^6^.

Hydrodistillation (HD) is one of the simplest conventional methods providing a high yield of essential oil, however, this method involves long extraction times with the possibility of decomposition, and sometimes the loss of heat-sensitive compounds during the extraction in addition to the possibility of its contamination with solvent or solvent residues^7^. Headspace is an alternative sampling method that is generally known as a vapor phase extraction involving the partitioning of the volatiles between the sample solution and the vapour phase ^8^. It is a very facile, sensitive, solvent-free sampling and concentration technique to capture the volatiles emitted by the living plant parts. Headspace traps generally show lower boiling constituents than the essential oil^9^, therefore it does not destroy the extracted oil by temperature^7^.

The family Zingiberaceae is very rich in essential oils, especially the rhizomes producing complex mixtures of essential oils responsible for their biological activities, distinctive aromatic profiles, and sweet-citrus, spicy, and camphorous characteristics^10,11^.

*Hedychium coronarium* J. Koenig (White Ginger Lily) is an erect perennial rhizomatous herb native to the mountains of Nepal and India. It is widely distributed in tropical and subtropical regions of India^12^. Traditionally, its various parts have tremendous medicinal properties in the Indian Ayurveda system as anti-rheumatic, tonic, and excitant^13^. In Chinese medicine, the rhizomes have been reported to be used as anti-diabetic and for headaches^12^. Its rhizomes were also used in tonsillitis, infected nostrils, tumors, and fever^13^. Recent studies have demonstrated that the ethanolic extract of its rhizomes exerted potential antiurolithiatic activity while the methanolic extract showed anti-inflammatory, anti-nociceptive, and CNS depressant activities^13^. Additionally, the essential oils extracted from its leaves, flowers, and rhizome were found to have molluscicidal, antimicrobial, anti-inflammatory, analgesic as well as antioxidant and antibacterial activities^12,13^.

Another Zingiberaceae plant is *Alpinia zerumbet* (Pers.) Burtt. et Smith. It is also known as shell ginger. *Alpinia zerumbet* is native to East Asia and widely cultivated in tropical and subtropical zones of the world^14^. It has been used as a flavor additive in food and traditional medicine for centuries. *A. zerumbet* seeds have been used as folk medicine in China and as a dietary supplement in Japan^15^, in addition, it has contributed significantly to prolonging human lives in the population of Okinawa^14^. Recent studies showed that the essential oil of fructus *A. zerumbet* has anti-oxidative, anti-inflammatory,^14^ and cardioprotective effects^16^ while the essential oil of the leaves has antimicrobial effect^17^. The methanolic extract of *A. zerumbet* flowers have anticancer effect^18^. Also, isolated 5,6-dehydrokawain and dihydro-5,6-dehydrokawain, from *A. zerumbet* have found to promote osteoblastic cell differentiation activities^19^.

Plants in the *Zingiberaceae* family contain complex mixtures of monoterpenes and sesquiterpenes that form their characteristic aroma and biological activities. Because extraction techniques differ in their selectivity, comparing hydrodistillation and headspace methods for two different species helps determine whether differences in chemical profiles arise from true biological variation in the plant—such as species, genotype, plant organ, growth conditions, or harvest stage—or from methodological artifacts. This comparison is particularly important for Zingiberaceae species because hydrodistillation involves prolonged heating and contact with water, which may modify their thermally sensitive volatile terpenoids through thermal degradation, hydrolysis of oxygenated terpenes, oxidation during heating, and the loss of highly volatile constituents. Whereas, headspace techniques better preserve the plant’s natural volatile emissions. Consequently, comparing these methods ensures more accurate chemical characterization and a more reliable interpretation of the biological activity associated with the detected compounds.

In this study, oils of leaves and rhizomes of *H. coronarium* and *A. zerumbet* were obtained by hydrodistillation and headspace methods then analyzed using GC/MS and compared for their constituents. Oils were also tested for their anticholinesterase and antioxidant activities. To the best of our knowledge, this is the first comparative study of the volatile constituents sampled by both headspace and hydrodistillation of leaves and rhizomes of *H. coronarium* and *A. zerumbet* cultivated in Egypt.

## Materials and methods

### Plant material

Leaves and rhizomes of *Hedychium coronarium* and *Alpinia zerumbet* were collected in August from the El Orman Botanical Garden, Egypt. Permission for collection was obtained from the garden administration, and all procedures were conducted in accordance with relevant institutional, national, and international guidelines for plant research. Both species were cultivated under semi-shaded conditions in soil characterized as an enriched alluvial clay loam amended with organic matter and sand and maintained under consistently moist but well-drained conditions. All plant materials were obtained from cultivated specimens, and no wild populations were disturbed. The species studied are not listed as endangered according to the International Union for Conservation of Nature (IUCN) Red List. The collection and use of plant materials adhered to ethical standards for biodiversity conservation, ensuring minimal environmental impact and sustainable use of biological resources. The plants were identified by Agricultural Engineer Mrs. Therese Labib, Senior Botanist, Orman Botanic Garden, Giza, Egypt. Voucher specimens (PHG-P-HC-473 and PHG-P-AZ-4) were deposited in the Herbarium of Pharmacognosy department, Faculty of Pharmacy, Ain Shams University.

### Distillation of essential oils

Fresh leaves and rhizomes of each plant were collected (500 g, each) and hydro-distilled separately using a Clevenger-type apparatus for 5 h. The oils were collected, dried over anhydrous sodium sulfate, and stored at 4 °C until analyzed. The essential oil yield was determined in terms of percentage by measuring the volume of the oil extracted per the weight of fresh plant material used.

### Headspace volatiles isolation

Three grams of the leaves and rhizomes of each plant were ground and placed inside 5 mL clear headspace glass vials, immediately sealed with silicone rubber septa. Volatile compounds were collected using dynamic headspace sorption then placed in a temperature-controlled tray at 80º C and introduced directly into the GC injector with a sample line and transfer line temperature of 150º C.

### Headspace GC/MS analysis

GC/MS analysis of the samples extracted by headspace was performed using Shimadzu GCMS-QP2020 (Koyoto, Japan) and Shimadzu HS-20. The GC was equipped with an Rtx-1MS fused bonded column (30 m × 0.25 mm i.d. × 0.25 µm film thickness) (Restek, USA) and a split–splitless injector. The initial column temperature was kept at 45 °C for 2 min (isothermal) and programmed to 300 °C at a rate of 5 °C/min and kept constant at 300 °C for 5 min (isothermal). The Helium carrier gas flow rate was 1.41 ml/min. The headspace condition was set as follows; the initial oven temperature was programmed at 80 °C, sample and transfer line temperature was programmed at 150 °C. The injection time was 1 min, and the needle flush time was 5 min. Mass spectra were recorded applying the following conditions: (equipment current) filament emission current, 60 mA; ionization voltage, 70 eV; ion source, 200 °C. A homologous series of *n*-alkanes (C_7_–C_30_) (Sigma Aldrich Chemical Company, USA) was injected under the same conditions described.

### GC/MS analysis of essential oils

The GC analysis of essential oils extracted by hydrodistillation was carried out using a Shimadzu GC MS-QP2010 (Koyoto, Japan) equipped with an Rtx-5MS column (30 m × 0.25 mm × 0.25 μm) (Restek, USA) and a split-splitless injector. Helium was used as the carrier gas with a flow rate of 1.41 mL/min). The initial column temperature was fixed at 45 ºC for 2 min (isothermal) and programmed to 300ºC at a rate of 5ºC/min, and kept constant at 300ºC for 5 min . The injector and detector temperatures were set at 250ºC and 300 °C, respectively. The ionsource and interface temperature was 200 °C and 280 °C, respectively. A volume of 1 µL of the diluted sample (1% v/v) in hexane was by injected in split mode with the split ratio 1:15. Results were processed using GCMSsolution Workstation Software for Gas Chromatography-Mass Spectrometry. Average areas under the peaks of three independent chromatographic runs were used for calculating the % composition of each component. A homologous series of *n*-alkanes (C_8_–C_28_) (Aldrich Chemical Company, USA) was injected under the same conditions described.

### Identification of Essential Oil Components

The essential oil components were identified by comparing their mass spectral data with the mass spectra in NIST 17 database. In addition, the Retention Index (RI) calculated relative to the homologous series of *n*-alkanes was compared to published RI values in Adams library (Adams, 2007) and online public databases^20,21^ ^22^ ^23^ ^24,25^. The relative content of individual components was calculated based on the relative GC peak areas (expressed as % from total peak areas).

### Multivariate analysis

The obtained data from GC/MS were subjected to chemometric analysis to find out the relations between different samples either different organs of the same species or to point out the similarities and differences among different species in addition to the extraction method (either hydrodistillation or headspace). Principal Component Analysis (PCA) was first applied as a primary step for the interpretation of the data to portray an overview of all sample discrepancies and to identify markers responsible for this dissimilarity. Hierarchal cluster analysis (HCA) was then applied to allow the clustering of different samples. The clustering pattern was constructed by the complete linkage method. PCA and HCA were achieved utilizing Unscrambler® X 10.4 from CAMO (Computer Aided Modeling, AS, Norway).

### Determination of anti-cholinesterase activity using colorimetric inhibition kit

The colorimetric inhibition kit was used to measure the ability of AChE enzyme to decompose the colored substrate and then measure the absorbance of the resulting yellow chromophore at 412 nm, followed by comparing it with reversible AChE inhibitors such as donepezil, tacrine, and rivastigmine^26^. The kit components include an AChE assay buffer, AChE substrate, Probe mix, and reversible inhibitors (Donepezil).

The essential oil was dissolved in DMSO (Merck) to obtain different concentrations. The sample mixture was further diluted with the AChE assay buffer then 10 μl of diluted test inhibitors were added into designated wells of a clear, flat-bottom 96-well plate. The reaction mixtures were transferred to the test wells of a 96-well microtiter plate. For each well, 40 μl reaction mix was prepared containing: 10 μl of diluted AChE substrate, 5 μl of probe mix, and 25 μl of AChE assay buffer were then added to test inhibitors (S), Inhibitor Control [IC], Enzyme Control [EC] and Background Control [BC] wells. The total reaction volume for each well was 200 μl. Enzyme Control (No Inhibitor) [EC] and Background Control [BC] were prepared by the addition of 100 μl of assay buffer to the designated well(s). Donepezil was used as a positive control. Finally, they were mixed well and incubated for 10–15 min at room temperature away from light. The absorbance (OD) was measured at 412 nm in kinetic mode for 40 min at room temperature. The inhibitory effect of the samples was calculated by comparison to the negative control:

$$\% {\text{ Relative Inhibition}} = \frac{{\left( {Slope of \left[ {EC} \right] - slope of\left[ S \right]} \right) }}{{Slope of \left[ {EC} \right]}} \times 100$$

$$\% {\text{ Relative Activity }} = \frac{Slope of \left[ S \right]}{{Slope of \left[ {EC} \right]}} \times 100$$

where EC is the enzyme control and S is the sample.

The inhibition of enzyme activity was expressed as IC_50_ (the concentration of the sample required to inhibit 50% of the enzyme), calculated by linear regression analysis.

### Oxygen radical antioxidant activity (ORAC):

The ORAC method using fluorescein as the fluorescent probe was previously described^27^. In our study, 25 μL of the diluted antioxidant Standard (Trolox) or samples were added to the 96-well microtiter plate and mixed with 150 μL of the 1X fluorescein solution then the plate was incubated at 37ºC for 30 min. 25 μL of 2,2-azobis(2-methylpropionamidine) dihydrochloride (AAPH; Sigma-Aldrich), the free radical initiator was then added into each well using either a multichannel pipette or a plate reader liquid handling system. The reaction mixture was mixed thoroughly by pipetting to ensure homogeneity. The reaction was performed at 37°C and fluorescence was recorded with an excitation wavelength of 480 nm and an emission wavelength of 520nm. Wells were read every 1–5 min. for 60 min. All reaction mixtures were prepared in triplicate. The ORAC values, expressed as μM Trolox equivalents (μM TE/g) were calculated by applying the following formula:

ORAC value (Sample) = 20 μM × 10 (dilution factor) = 200 μM TE = 200 μMole TE/L.

#### Statistical methods

The bioactivity assays were performed in three replicates and results are presented as Mean ± SD. Linear regression was used to calculate IC_50_ for % ACHE inhibition against the different concentrations of test samples by means of Excel Program 2015**.**

## Results and discussion

### Comparative analysis of the essential oils of *H. coronarium* leaves

For both plant parts, analysis of the hydrodistilled and headspace volatiles was carried out to obtain a more accurate description of the volatile oil chemical composition. The relative contents of the identified volatile compounds were expressed as the percentage of peak area relative to the total area of all peaks (Table 1).

**Table 1:** Chemical composition of essential oils extracted by hydrodistillation and headspace from both leaves and rhizomes of *H. coronarium* and *A. zerumbet* cultivated in Egypt.

	Compound	RI Calculated	RI Reported	Relative content [%]^a^							
				*H. coronarium* Le		*H. coronarium* Rh		*A. zerumbet* Le		*A. zerumbet* Rh	
				HD	HS	HD	HS	HD	HS	HD	HS
1	Hexenal	827	827	–	4.99	–	–	–	6.85	–	–
2	*α*-Thujene	926	930	–	0.25	0.31	0.45	3.9	4.37	1.39	6.10
3	*α*-Pinene	932	932	6.63	26.19	7.32	10.33	1.96	1.53	1.59	4.36
4	Camphene	939	939	–	0.22	0.66	0.57	0.27	0.36	0.73	0.69
5	Sabinene	965	975	–	1.63	–	23.87	6.54	21.62	7.79	6.17
6	***β*-Pinene**	969	969	1.13	**48.94**	23.80	0.43	4.05	1.12	5.55	12.27
8	*β*-Myrecene	986	986	8.90	0.17	1.50	–	0.71	1.56	1.03	1.60
9	*α*-Phellandrene	999	999	–	–	3.39	0.62	0.17	–	0.61	0.62
10	*p*-Mentha-1 (7), 8-diene	999	1004	–	–	0.74	–	–	–	–	–
11	*α-*Terpinene	1006	1006	–	–	0.41	0.12	2.44	2.52	5.51	6.29
12	*δ*-3-Carene	1007	1007	–	–	–	0.27	–	–	–	–
13	*p*-Cymene	1014	1014	–	1.34	–	1.55	7.8	5.45	1.89	10.42
14	**1,8-Cineole (Eucalyptol)**	1021	1021	3.25	10.07	**41.69**	**58.41**	19.64	**24.79**	**20.78**	**23.63**
15	Sylvestrene	1024	1024	–	1.42	–	–	–	–	–	–
16	(*Z*)-*β*-Ocimene	1037	1037	0.09	–	–	–	–	–	–	–
17	(*E*)-*β*-Ocimene	1038	1037	–	–	0.05	–	–	–	–	–
18	*δ*-Terpinene	1049	1051	–	–	2.08	–	–	–	–	–
19	γ-Terpinene	1052	1052	0.18	–	0.26	0.52	13.98	8.49	11.27	13.15
20	*cis*-Sabinene hydrate	1055	1054	–	–	–	–	–	3.27	–	0.72
21	*p*-Mentha-2, 4(8)-diene	1076	1088	0.09	–	0.60	0.16	–	–	1.47	–
22	*trans*-Sabinene hydrate	1084	1087	–	–	–	–	–	2.87	–	0.70
23	Terpinolene	1088	1088	–	–	–	–	1.45	1.14	2.36	2.59
24	Linalool	1089	1089	0.27	–		–	–	0.67	–	–
25	*endo*-Fenchol	1103	1103	–	–	0.09	–	–	–	0.16	–
26	Thujone	1106	1106	–	–	0.02	–	–	–	–	–
27	*cis*-*p*-Ment-2-en-1-ol	1108	1108	–			–	–	0.49	–	–
28	*α*-Campholenal	1115	1111	0.05	0.62	0.09	–	–	–	–	–
29	Thujen-3-ol	1123	1132	–	0.66	–	–	–	–	–	–
30	*trans*-*p*-Ment-2-en-1-ol	1124	1127	–	–	–		–	0.24	–	–
31	*trans*-Pinocarveol	1128	1129	0.20	–	–	–	–	–	–	–
32	Camphor	1137	1137	–	–	0.11	–	–	–	0.16	–
33	Ethanone, 1-(1,4-dimethyl-3-cyclohexen-1-yl)	1145	1152	–	–	0.03	–	–	–	–	–
34	Pinocarvone	1151	1164	0.23	0.67	0.19	–	–	–	–	–
35	Borneol	1155	1155	0.14	–	–	–	–	–	0.92	–
36	Isoborneol	1156	1156	–	–	1.34	0.16	–	–	–	–
37	*trans*-Pinocamphone	1163	1163	–	–	0.08	–	–	–	–	–
38	Terpinen-4-ol	1168	1168	1.11	0.55	4.51	–	**24.72**	10.3	20.75	9.52
39	Myrtenal	1168	1168	–	0.72	0.26	–	–	–	–	–
40	*γ*-Terpineol	1173	1170	–	–	–	1.05			–	–
41	*α*-Terpineol	1182	1182	0.78	–	4.42	–	1.75	0.61	2.15	–
42	Myrtenol	1185	1185	0.61	0.47		–	–		–	–
43	Ascaridole	1212	1221	–	–		–	–	0.91	–	–
44	Isobornyl formate	1216	1217	–	–	0.02	–	–	–	–	–
45	Citronellol	1217	1217	0.06	–		–	–	–	–	–
46	*α*- Fenchyl acetate	1222	1223	–	–	–	–	–	–	3.66	––
47	Carvone	1234	1234	0.06	–	–	–	–	–	–	–
48	Linalool acetate	1243	1244	0.33	–	–	–	–	–	–	–
49	Isobornyl acetate	1275	1275	0.11	–	–	–	–	–	–	–
50	Bornyl acetate	1276	1276	–	–	0.11	–	–	–	–	–
51	*δ*-Elemene	1326	1338	0.31	–	–	–	–	–	–	–
52	*α*-Terpinyl acetate	1338	1336	0.24	–	0.56	–	–	–	–	–
53	Citronellyl acetate	1341	1352	0.14	–	–	–	–	–	–	–
54	*β*-Elemene	1380	1390	0.23	–	–	–	–	–	–	–
55	**Caryophyllene**	1413	1413	**45.24**	0.20	–	0.17	2.55	0.39	1.25	1.16
56	Aromadendrene	1438	1438	0.06	–	–	**–**	–	**–**	–	–
57	*α*-Humulene	1444	1444	2.90	–	0.10	–	0.32	–	–	–
58	Germacrene D	1471	1471	0.16	–	–	–	–	–	–	–
59	Valencene	1505	1496	–	–	0.04	–	–		–	–
60	*δ*-Amorphene	1513	1512	0.13	–	–	–	–		–	–
61	*trans*-Calamenene	1514	1515	–	–	0.02	–	–		–	–
62	*trans*-Muurola-4(14), 5-diene	1519	1519	–	–	–	–	0.26	–	–	–
63	(*E*)-Nerolidol	1550	1554	0.43	–	–	–	–	–	–	–
64	Longipinocarvone	1567	1569	2.30	–	–	–	–	–	–	–
65	Caryophyllene oxide	1576	1576	12.49	–	0.46	–	1.87	–	0.34	–
66	*cis*-*β*-Elemenone	1584	1586	0.12	–	–	–	–	–	–	–
67	Spathulenol	1590	1619	0.52	–	–	–	–	–	–	–
68	Carotol	1604	1600	–	–	–	–	–	–	0.29	–
69	Muurola-4,10(14)-dien-1*β*-ol	1619	1631	0.65	–	–	–	–	–	–	–
70	Caryophylla-4(12),8(13)-dien-5*α*-ol	1624	1627	0.74	–	–	–	–	–	–	–
71	Caryophylla-4(12),8(13)-dien-5*β*-ol	1628	1640	2.73	–	–	–	0.16	–	–	–
72	*τ*-cadinol	1630	1630	0.21	–	0.29	–	–	–	–	–
73	Cubenol	1645	1645		–	0.05	–	–	–	–	–
74	Alloaromadendrene epoxide	1649	1646	0.30	–	–	–	–	–	–	–
75	14-Hydroxy-9-epi-(*E*) Caryophyllene	1662	1664	1.41	–	–	–	–	–	–	–
76	Longifolol	1736	1717	0.56	–	–	–	–	–	–	–
77	Ambrial	1802	1806	1.01	–	0.08	–	–	–	0.20	–
78	(*E*)-15,16-Dinorlabda-8(17),11-dien-13-one	1976	1994	0.21	–	–	–	–	–	–	–
79	(6*Z*, 10*Z*)-Pseudo phytol	1991	1988	0.07	–	–	–	–	–	–	–
80	6Z, 10*E*-Pseudo phytol	2058	2031	0.06	–	–	–	–	–	–	–
81	Manool	2073	2057	0.15	–	–	–	–	–	–	–
82	2-Hexadecen-1-ol, 3,7,11,15-tetramethyl	2094	2114	0.14	–	–	–	–	–	–	–
83	*n*-Tricosane	2275	2300	–	–	0.02	–	–	–	–	–
84	Pentacosane	2469	2500	–	–	0.03	–	–	–	–	–
85	Hexacosane	2668	2600	–	–	0.05	–	–	–	–	–
86	Nonacosane	2853	2900	–	–	0.04	–	–	–	–	–
87	Triacontane	3026	3000	–	–	0.02	–	–	–	–	–

^a^Values are expressed as relative area percentages; The major components are highlighted in bold. (Values expressed as relative area percentages to the total identified components).

*Tentatively identified according to the mass spectrum (MS) and by comparison of RI with the literature.

The oil obtained by hydrodistillation of the fresh leaves was light yellow colored with a characteristic aromatic odor; the average yield was 0.12% (*v/w*). The composition of the essential oil extracted employing both techniques was compiled in Table 1. Some components, like camphene, sabinene, sylvestrene, myrtenal, and thujen-3-ol were identified only by the HS method.

Forty-seven compounds representing 97.73% of the leaf oil were identified in the hydrodistilled leaf oil compared to sixteen compounds representing 99.17% (Table 1) identified in headspace aroma. Sesquiterpenes hydrocarbon dominated the oil content obtained by hydrodistillation (49.03%) where *β-*caryophyllene **(45.24%)** was the major component and caryophyllene oxide (12.49%) represented the major oxygenated sesquiterpene (23.47%). Additionally, monoterpene hydrocarbon constituted 16.89%, while oxygenated monoterpenes amounted to 7.38% where 1,8-cineole (3.25%) was the major representative. Diterpenoids represented 0.49% whereas the identified labdane diterpenoids constituted 0.36%. The high content of caryophyllene and caryophyllene oxide was in agreement with a previous study^28^, while in other studies oxygenated monoterpenes as 1,8-cineole dominated the oil content^29^ or monoterpenes as *β*-pinene, and *α*-pinene were the major constituents^30^. Differences in the composition of the oil may be due to many factors such as collection and extraction methodology, physiological variations or genetic factors, and environmental conditions such as seasonal variations and the type of soil^31^ ^32^.

On the other hand, monoterpenes dominated the oil content (85.21%) obtained by headspace where *β***-**pinene (48.94%) was the major component followed by *α*-pinene (26.19%.) The oxygenated monoterpenes came in the second order (13.76%) and were represented by 1,8-cineole (10.07%), while** s**esquiterpenes were identified in small amounts as caryophyllene (0.2%).

### Comparative analysis of the essential oils of *H. coronarium* rhizomes

The essential oil obtained by hydrodistillation of rhizomes produced bright yellow-colored oil, with a strong characteristic aromatic odor. The yield was 0.16% (v/w). Thirty nine components constituting 95.84% compared to fifteen compounds representing 98.68% of the oil were identified from the rhizome essential oil by hydrodistillation and headspace respectively (Table 1). Oxygenated monoterpenes dominated the identified classes by both methods (53.52% and 59.62%) where 1,8-cineole (Eucalyptol) (41.69% and 58.41%) was the major component followed by monoterpenes hydrocarbon (39.08% & 38.89%) with *β*-pinene (23.8%) as the major component by hydrodistillation in agreement with previous studies^30^ ^28^ ^33^**.** The prevalence of eucalyptol is noteworthy, as it has been reported to exert anti-inflammatory, antioxidant, and neuroactive properties. It enhanced stress resistance and alleviated A*β*-induced paralysis. Additionally, it showed an anti-alzheimer’s disease effect^34^.

Another study showed trans-meta-mentha-2,8-diene, linalool as the major identified constituents^35^. Comparatively, Sabinene (23.87%) and *α*-pinene (10.33%) presented in higher concentrations by headspace.

### Comparative analysis of the essential oils of *A. zerumbet* leaves

The oil obtained by hydrodistillation of the fresh leaves of *A. zerumbet* is dark yellow colored with a strong characteristic odor; the average yield was 0.4% v/w. The composition of the oil obtained from both methods was compiled in Table 3. Nineteen compounds (94.54%) compared to twenty-one compounds (99.53%) were identified from hydrodistillation and headspace respectively. Oxygenated monoterpenes dominated the oil content (46.11%) obtained by hydrodistillation where terpinene-4-ol (24.72%) was the major compound followed by 1,8-cineole (19.64%). Monoterpene’s hydrocarbon came in the 2nd order representing (43.27%) where *γ*-terpinene was the major constituent (13.98%). A previous study has also shown that the hydrodistilled oil of *A. zerumbet* leaves cultivated in Egypt was mainly composed of terpinen-4-ol (17.3%) followed by 1,8-cineole (14.4%)^36^. Other studies have also reported that terpinen-4-ol was the major constituent in the essential oil of *A. zerumbet* leaves in other countries^32^^,^^37,38^.

On the other hand, monoterpene hydrocarbons dominated the oil content obtained by headspace (48.16%) whereas sabinene (21.62%) was the major representative. Oxygenated monoterpenes accounted for 44.15% represented by 1,8-cineole (24.79%) and terpinen-4-ol (10.3%) where 1,8-cineole is (24.79%) was the major constituent in the leaf aoil obtained by headspace.

### Comparative analysis of the essential oils of *A. zerumbet* rhizomes

The oil obtained by hydrodistillation of the fresh rhizomes of *A. zerumbet* is dark yellow colored with a strong characteristic odor; the average yield was 0.1% v/w. The composition of the oil obtained from both methods was compiled in Table 4. Twenty-three compounds (91.85%) compared to sixteen constituents (100%) were identified from *A. zerumbet* rhizomes by hydrodistillation and headspace, respectively. Similar to the leaves, oxygenated monoterpenes represented the major identified class obtained by hydrodistillation (48.58%) but 1,8-cineole (20.78%) was the major compound followed by terpinen-4-ol (20.75%). Monoterpene hydrocarbons represented 41.19% exemplified by γ-terpinene (11.27%). Those results were in agreement with a previous study^39^ whereas another study showed terpinen-4-ol as the major constituent followed by *α*-terpinene^38^. Other studies reported that terpinen-4-ol was the major constituent in the rhizome oil of *A. zerumbet* growing in Egypt^40^. Even though monoterpene hydrocarbons (64.26%) dominated the oil content obtained by headspace where γ-terpinene (13.15%) and *β*-pinene (12.27%) were the major representatives. Oxygenated monoterpenes came in the second order representing 34.57% where 1,8-cineole (23.63%) was the major component.

### GC/MS-based chemometric analysis

Both principal component analysis (PCA) and hierarchal cluster analysis (HCA) were applied as chemometric tools to explore their abilities to discriminate between various samples, as well as to identify any significant correlation between them. PCA was applied as an initial phase to reduce the dimensionality of the multiple data sets in addition to removing the redundancy in the variables, utilizing raw data (Peak area % for each compound).

**Fig. 1:**
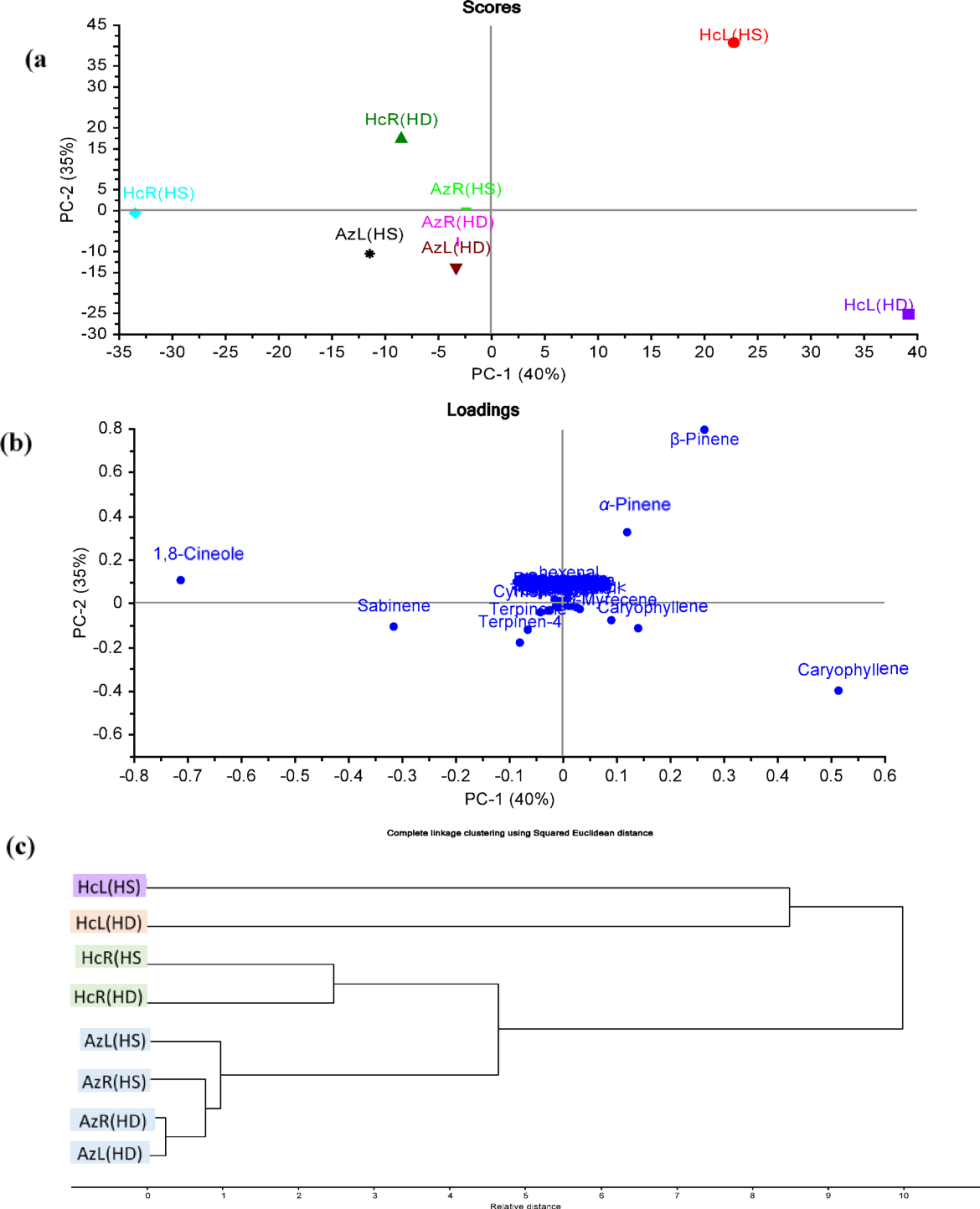
(**a**) PCA Score plot, (**b**) PCA loading plot, (**c**) HCA based on GC/MS identification of the chemical compositions of the essential oils extracted by both hydrodistillation and headspace from leaves and rhizomes of *H. coronarium* and *A. zerumbet* cultivated in Egypt.

First PCA was applied to both *H. coronarium* and *A. zerumbet* leaves and rhizomes essential oils extracted by both hydrodistillation and headspace. Figure 1a represents the PCA score based on GC/MS identification of the chemical compositions of *H. coronarium* and *A. zerumbet* leaves and rhizomes essential oils extracted by both hydrodistillation and headspace revealing a significant statistical segregation among various samples. PCA score plot (Fig. 1a) explained about 75% of the dataset variation by the first two PCs, where PC1 accounted for 40% and PC2 for 35% of the variance. Various samples were segregated into four main groups on four single quadrants. It was observed that both oils obtained from *H. coronarium* leaves by hydrodistillation and headspace were located on the positive score values (right side of PC1) on the lower and upper quadrants respectively. However, all other samples were positioned on a negative score plot (negative side of PC1). Oils of *H. coronarium* rhizomes were on the upper quadrant (negative PC1 and positive PC2), completely segregated away from each other. On the other side, oils of *A. zerumbet* leaves and rhizomes, extracted by both methods were grouped on the lower quadrant (negative PC1 and PC2) related to each other. Figure 1b represents the loading plot of PCA with significant markers responsible for sample segregation. *β*-pinene and caryophyllene were the key components responsible for the separation of oils of *H. coronarium* leaves obtained by headspace and hydrodistillation extraction methods respectively. Regarding the oil of *H. coronarium* rhizomes extracted by hydrodistillation, cineole was the main compound accounting for its separation. Furthermore, HCA was employed as an unsupervised pattern recognition method to endorse the results of PCA. Figure 1c displayed the HCA dendrogram, and confirmed the segregation of various samples into four main clusters. Clusters I, and II presented the separation of oils of *H. coronarium* leaves by headspace and hydrodistillation respectively. Cluster III included the essential oils of *H. coronarium* rhizomes showing 2 subclusters for different extraction methods. Regarding the oils of *A. zerumbet* leaves and rhizomes, they were grouped in cluster IV confirming the results of PCA.

**Fig. 2:**
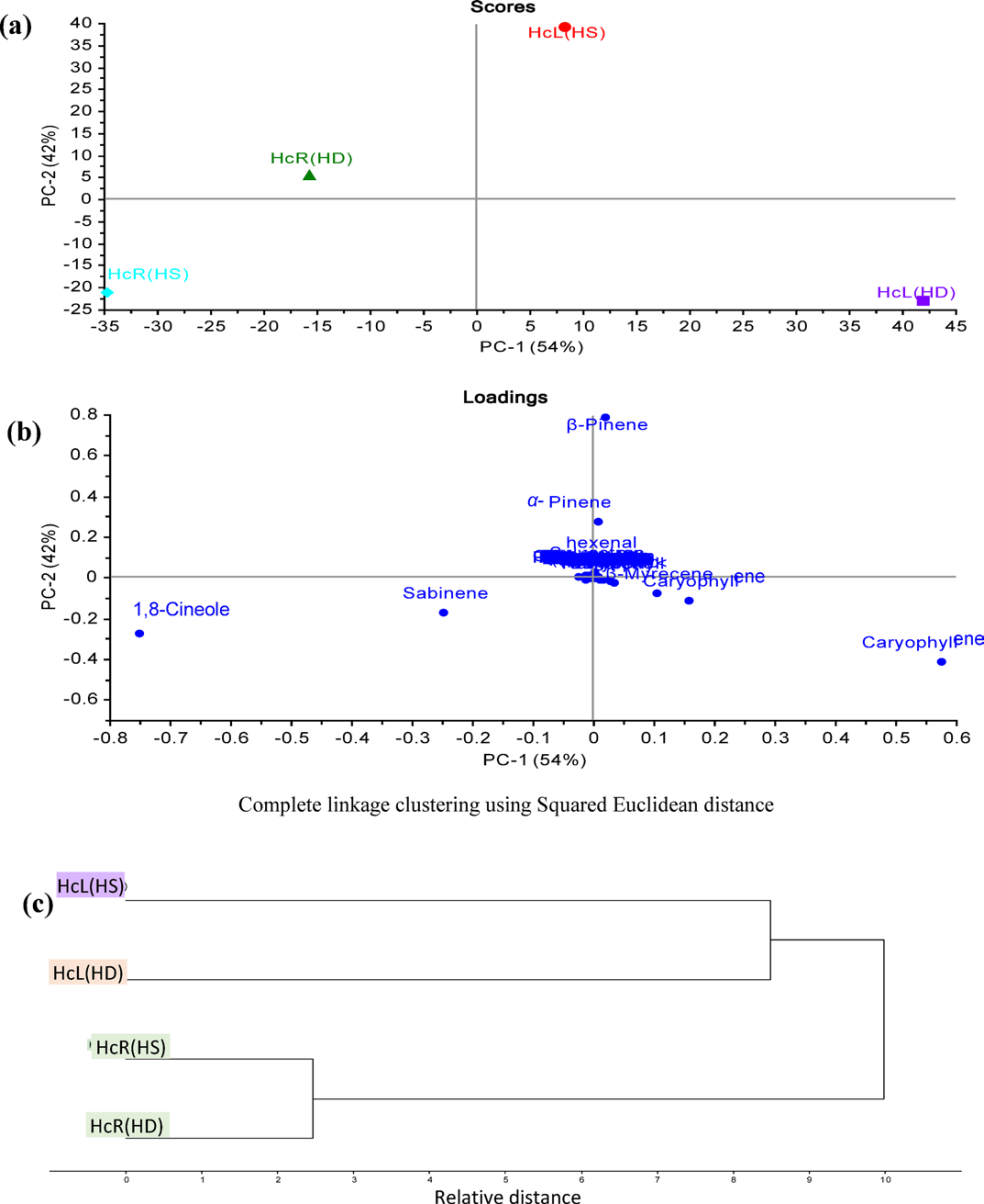
(**a**) PCA Score plot, (**b**) PCA loading plot, (**c**) HCA based on GC/MS identification of the chemical compositions of the essential oils extracted by hydrodistillation and headspace from leaves and rhizomes of *H. coronarium* cultivated in Egypt.

PCA was also applied to oils obtained from both leaves and rhizomes of *H. coronarium* including different extraction methods, Fig. 2a shows a PCA score plot, confirming results previously obtained where the same pattern was obtained with complete segregation of all samples. Moreover, the PCA loading plot (Fig. 2b) displayed the same discriminating markers for *H. coronarium* segregation, in addition to the sabinene compound that separated *H. coronarium* rhizome oil extracted by the hydrodistillation method. HCA (Fig. 2c) confirmed the results of PCA.

PCA was utilized to segregate closely related oils of *A. zerumbet* leaves and rhizomes. (Figure S9a, supplementary data) displayed the PCA score plot, where different *A. zerumbet* oil samples were grouped based on the extraction method. The oils of *A. zerumbet* leaves and rhizomes extracted by hydrodistillation were placed on positive PC1 in the same quadrant, however, completely separated from each other. On the other side samples extracted by headspace, leaves were positioned on the lower quadrant (negative PC1 and PC2). On the contrary, oils of *A. zerumbet* rhizomes extracted by headspace were positioned on the upper quadrant. By examination of the loading plot (figure S9b, supplementary data), showed that *β*-pinene and sabinene were the key markers accounting for *A. zerumbet* rhizomes and leaves separation by headspace. Concerning extraction by hydrodistillation, terpenine-4-ol was identified as the main component responsible for the observed pattern. HCA dendrogram (figure S9c, supplementary data) grouped samples into three main clusters verifying the results of PCA.

By employing PCA for both oils from the leaves of *H. coronarium* and *A. zerumbet*, the PCA score plot (figure S10a, supplementary data) revealed that both extraction methods (hydrodistillationand headspace) exhibited no differences regarding *A. zerumbet* chemical composition as both were superimposed over each other in the same quadrant. In contrast to the oils of *H. coronarium* leaves*,* they were segregated from each other in two different quadrants. Loading plot figure S10b, supplementary data) displayed the same markers as that observed in Fig. 1b for HcL (HS) and HcL (HD) separation. HCA dendrogram (figure S10c, supplementary data) revealed the closeness of the essential oils of *A. zerumbet* leaves by different extraction methods.

Results of PCA (figure S11a supplementary data) for the oils of both *H. coronarium* and *A. zerumbet* rhizomes were similar to that obtained for the oils of leaves to some extent, where oils of *A. zerumbet* rhizomes obtained by different extraction methods were positioned on the same quadrant revealing the resemblance of their chemical composition. However, oils of *H. coronarium* rhizomes were located on two separate quadrants. From the loading plot (figure S11b supplementary data), terpeneine and terpinen-4-ol accounted for the segregation of the oils of *A. zerumbet* rhizomes. The same results were obtained for HCA (figure S11c supplementary data).

### In Vitro Antioxidant activity

Oxidants are oxidizing agents or free radicals which usually have unpaired electrons such as hydroxyl, alkoxyl, or reactive oxygen species. They are very reactive and can attack molecules. Free radicals may cause oxidative stress which causes the depletion of the immune system’s antioxidants, and a change in the patterns of gene expression causing abnormal proteins and resulting in degenerative diseases and aging^41^. Antioxidants can prevent the oxidation process indirectly or directly either by hydrogen-atom transfer (an antioxidant compound quenches free-radical species by donating hydrogen atoms) or by the single-electron transfer (an antioxidant transfers a single electron to aid in the reduction of potential target compounds)^42^. Therefore, antioxidant activity is found to be highly correlated with the maintenance of good health in humans, and hence several methods were developed for the quantification of antioxidant activity of edible flowers’ extracts. These methods may be categorized based on the chemistry of the reactions involved such as the methods that pertain to the mechanisms of hydrogen-atom transfer including oxygen radical absorbance capacity (ORAC) assay^42^.

### Oxygen radical activity capacity (ORAC) assay

The Oxygen Radical Absorbance Capacity (ORAC) assay is a method that measures the antioxidant capacity of a substance. It depends on measuring a fluorescent signal from a probe that is quenched in the presence of Reactive Oxygen Species (ROS). These ROS are generated upon thermal degradation of (AAPH (2,2′-azobis(2-methylpropionamidine) dihydrochloride) which is a ROS generator. AAPH is reactive with water and lipid-soluble substances, so ORAC can measure the total antioxidant potential^43^.

The subsequent addition of an antioxidant absorbs the generated ROS, allowing the fluorescent signal to persist. In this method, Trolox® (6-hydroxy-2, 5, 7, 8-tetramethylchromane-2-carboxylic acid) is used as a standard antioxidant by which all unknown antioxidants are compared^44^.

ORAC can be used to determine the total antioxidant capacity of biological fluids, cells, and tissue. It can also be used to assay the antioxidant activity of naturally occurring or synthetic compounds for use as dietary supplements, topical protection, and therapeutics.

ORAC assay is more suitable to mimic the inactivation of free radicals and to determine the radical scavenging activities in human cells as the antioxidant capacity in the ORAC assay is determined after a warm-up phase, in which the 96-well plate is heated to 37°C in a fluorometer which is the temperature of the human cells. There is also a continuous release of free radicals over 90 min. Therefore, those antioxidants that require a longer reaction time for inactivation of the reactive species get enough time for this operation^45^.

**Fig. 3:**
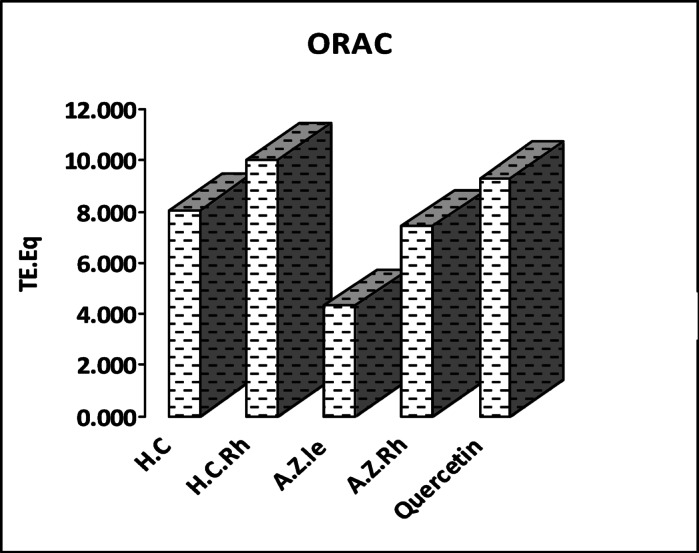
Oxygen radical absorbance capacity ORAC of the studied essential oils of *H. coronarium and A. zerumbet* expressed in µmol Trolox equivalent per g oil.

The antioxidant activity of the essential oils obtained from leaves and rhizomes of *H. coronarium* and *A. zerumbet* was determined in this study using ORAC assay. The results were compared to quercetin (Fig. 3).

From the previous Fig. 3, it was found that the essential oil of *H. coronarium* rhizomes showed the strongest ORAC antioxidant activity (10.06 ± 0.16 TE µM/L) compared to quercetin (9.317 ± 0.23 TE µM/L) followed by the oil of its leaves 8.05 ± 0.25 TE µM/L. ORAC antioxidant activity of the essential oils of *A. zerumbet* organs come in the second order where rhizomes oil showed higher antioxidant activity 7.42 ± 0.29 TE µM/L than the leaves oil 4.32 ± 0.29 TE µM/L. To the best of our knowledge, this is the first study of the ORAC antioxidant activity of the essential oils of leaves and rhizomes of *H. coronarium* and *A. zerumbet* growing in Egypt.

### Acetylcholine esterase inhibition activity of *H. coronarium* essential oils

Acetylcholinesterase (AChE) is the predominant cholinesterase in the brain that is involved in the termination of impulse transmission by rapid hydrolysis of the important neurotransmitter ACh. Disturbances in its levels are associated with the pathogenesis and progression of many neurodegenerative diseases and depressive disorders. Acetylcholine esterase has common functions with most of the described disorders such as participation in oxidative stress, inflammatory response, role in apoptosis, and role in adhesion of pathological proteins^46^.

Anticholinesterase or AChE inhibitors currently have several properties that may help to slow disease progression. It is commonly believed that they act only symptomatically and not causally. The AChE is found to be linked with the pathogenesis and progression of Alzheimer’s disease (AD). Hence, inhibiting this enzyme activity represents an important therapeutic strategy for treating some symptoms of this disease by enhancing the ACh level in the brain. Anticholinesterase became the main class of drugs currently used for the treatment of AD dementia phase, leading to an increase in both the level and duration of the neurotransmitter action and improving cholinergic function in the brain^44^.

Clinically relevant AChEI, such as donepezil, rivastigmine, tacrine, and galanthamine, are commonly approved for the treatment of AD and applied in neurodegenerative disorders treatment but they have limitations for clinical use due to their short half-lives and antagonistic side effects. Therefore, looking for new AChEIs with higher efficacy and safety from alternative sources such as natural products is the goal of nowadays investigators^26^.

The acetylcholine esterase inhibitory activity of essential oils from leaves and rhizomes of *H. coronarium* and *A. zerumbet* was compared to that of donepezil, tacrine, and rivastigmine which inhibited the acetylcholinesterase with IC_50_ = 0.134 ± 0.007, 0.19 ± 0.01 and 3.581 ± 0.191 respectively. *A. zerumbet* rhizomes essential oil showed the strongest activity against acetylcholine esterase with IC_50_ = 0.536 ± 0.022 µg/ml, followed by *H. coronarium* leaves essential oil with IC_50_ = 1.033 ± 0.042 µg/ml. A previous study testing the anticholinesterase activity of *H. gardnerianum* leaf oil from four different places showed potent activity with IC_50_ values ranging from 1.03** ± **0.14 to 1.37 ± 0.27 mg/ml^47^.

The strong anticholinesterase activity of the *A. zerumbet* rhizome oil may result from a synergistic interaction among its major constituents, particularly, 1,8-cineole and terpinene-4-ol rather than the activity of single isolated compounds^48^. Both compounds have been associated with strong and moderate enzyme inhibition, respectively in addition to neuroactive properties; however, their individual effects are generally weaker than those observed for the whole essential oil^49^.

**Fig. 4:**
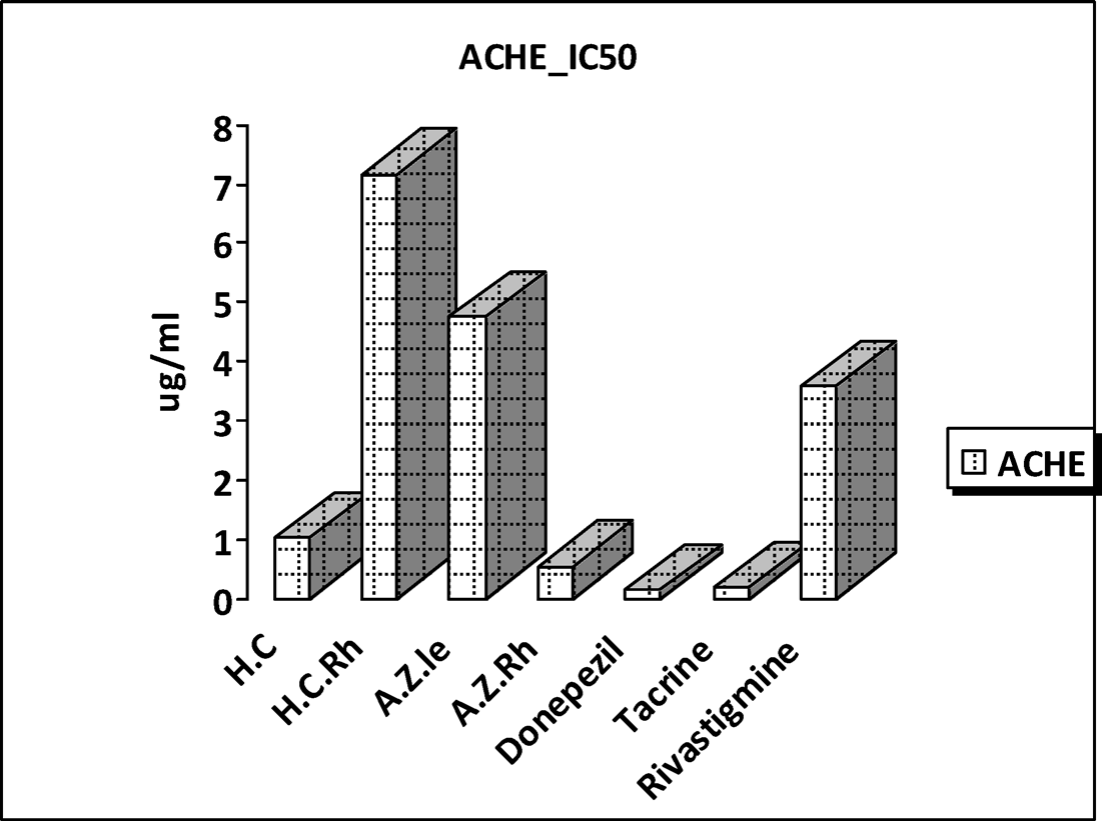
Acetylcholine esterase inhibitory activity of the essential oils of *H. coronarium and A. zerumbet* and the inhibitor controls.

The weakest activity was shown by *H. coronarium* rhizomes essential oil with IC_50_ = 7.166 ± 0.293 µg/ml (Fig. 4). Similar results were previously reported in *H. flavum* rhizome essential oil which showed weak anticholinesterase activity IC_50_ = 4.84 ± 0.36 mg/ml^50^, while the essential oil *H. puerense* rhizome showed a significant AChE inhibitory activity with lower IC_50_ = 0.94 ± 0.02 mg/mL^51^. No studies were traced regarding the anticholinesterase activity of essential oils of leaves and rhizomes of both *H. coronarium* and *A. zerumbet* in Egypt.

## Conclusion

Findings from this study support that different extraction methods significantly influence the volatile profile, where using the non-destructive headspace technique provides a much real representation of the plant’s native aromatic composition, especially for the highly volatile and thermally sensitive constituents. Chemometric analysis confirmed clear method-dependent discrimination, highlighting the importance of extraction strategy in accurate phytochemical characterization. The strong anticholinesterase activity of *A. zerumbet* rhizomes oil and the antioxidant activity of *H. coronarium* leaves oil indicate their potential as natural multi-target agents relevant to treat Alzheimer’s disease by increasing the levels of acetylcholine in cholinergic neurons, while simultaneously preventing further degradations caused by radical oxygen species. Future in vivo studies are recommended to validate these activities and explore their neuroprotective mechanisms.

## Supplementary Information

Supplementary Information.

## Data Availability

The datasets generated and analyzed during the current study are all available upon request.
