# Supplementary material for: Comparative analysis of volatile composition and anticholinesterase activity of Egyptian Hedychium coronarium and Alpinia zerumbet using chemometric assessment of extraction techniques
Source: Sci Rep. 2026 May 15;16:15209. doi: 10.1038/s41598-026-51750-1 (PMC13179332; doi:10.1038/s41598-026-51750-1)
Supplement: Supplementary file 1 — Supplementary Information. [file 41598_2026_51750_MOESM1_ESM.pdf]

SUPPLEMENTARY MATERIAL

"Comparative Volatile Profiling and Bioactivity of Egyptian *Hedychium coronarium* and *Alpinia zerumbet*: A Chemometric Evaluation of Extraction Techniques and Anti-cholinesterase Potential."

Esraa Adel Shahat [a)], Iriny M. Ayoub [b)], Riham O. Bakr [a)], Haidy A. Gad [b)], Omayma A. Eldahshan [b), c)*], Abdel Nasser B. Singab [b), c)*]

[a)] Pharmacognosy Department, Faculty of Pharmacy, October University for Modern Sciences and Arts (MSA), Giza, Egypt.

[b)] Pharmacognosy Department, Faculty of Pharmacy, Ain Shams University, Cairo, Egypt

[c)] Centre for Drug Discovery Research and Development, Ain Shams University, Cairo 11566, Egypt.

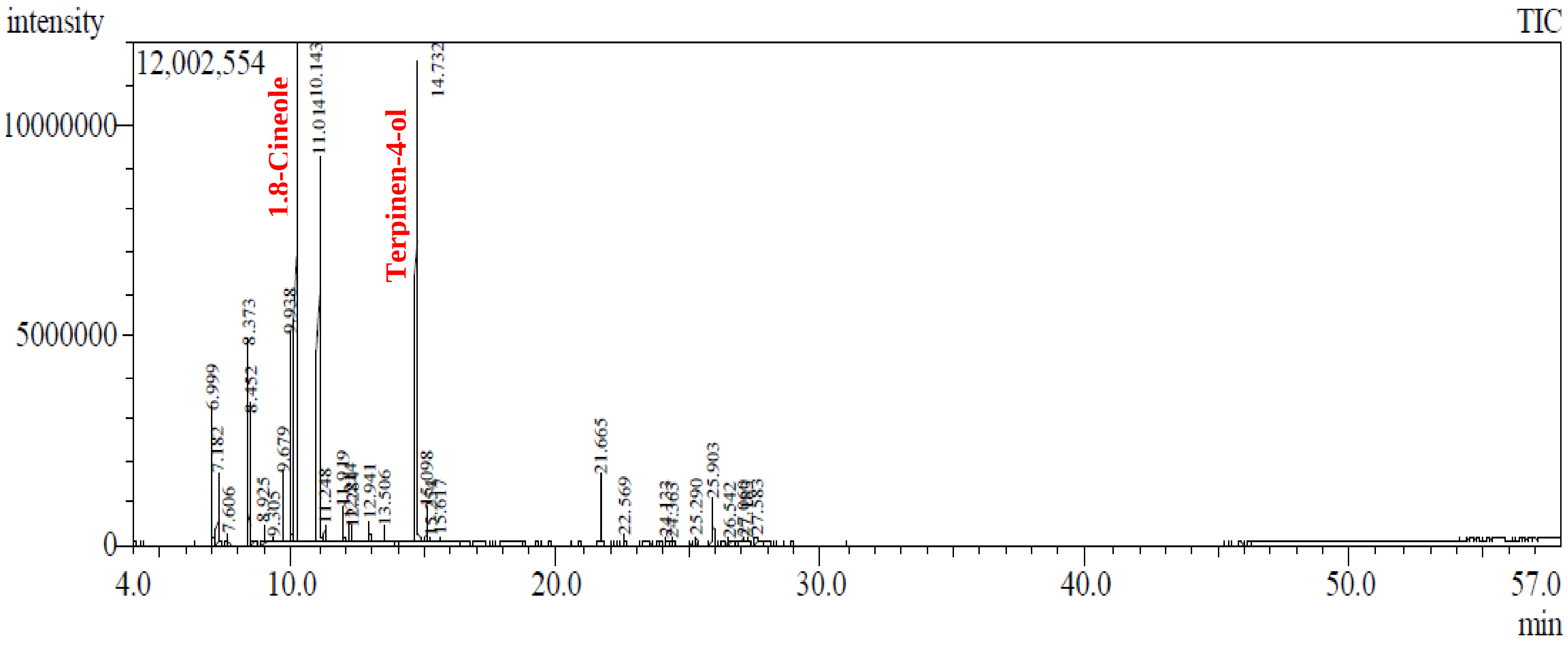

Figure S1: Total Ion Chromatogram of the Essential Oil Extracted from *Alpinia zerumbet* Leaves by Hydrodistillation

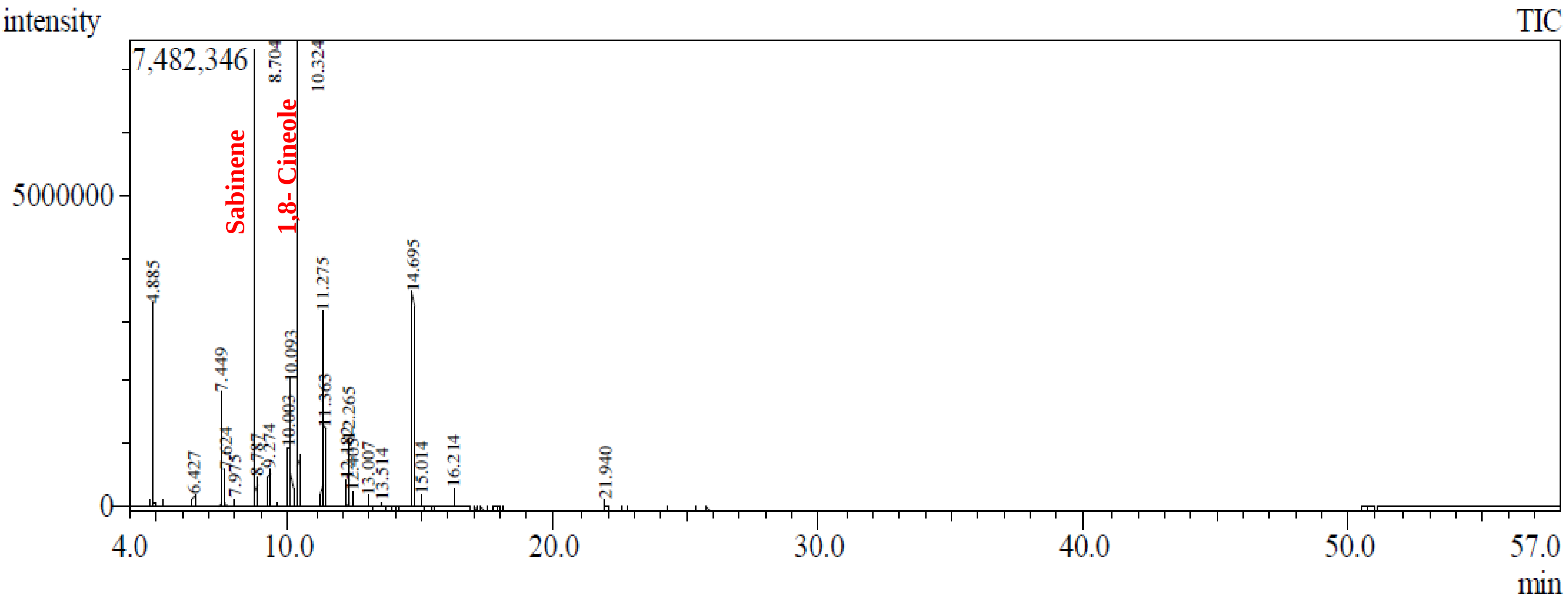

Figure S2: Total Ion Chromatogram of the Essential Oil Extracted from *Alpinia zerumbet* Leaves by Headspace

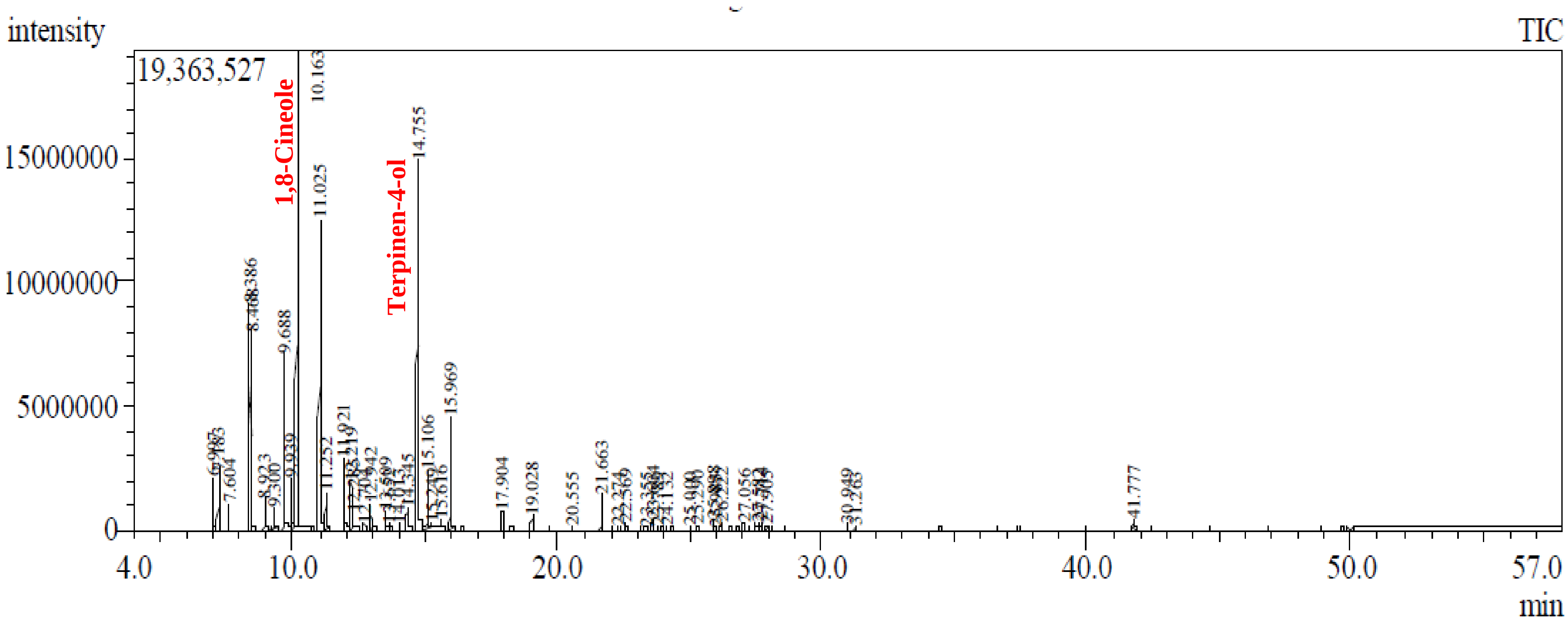

Figure S3: Total Ion Chromatogram of the Essential Oil Extracted from *Alpinia zerumbet* Rhizomes by Hydrodistillation

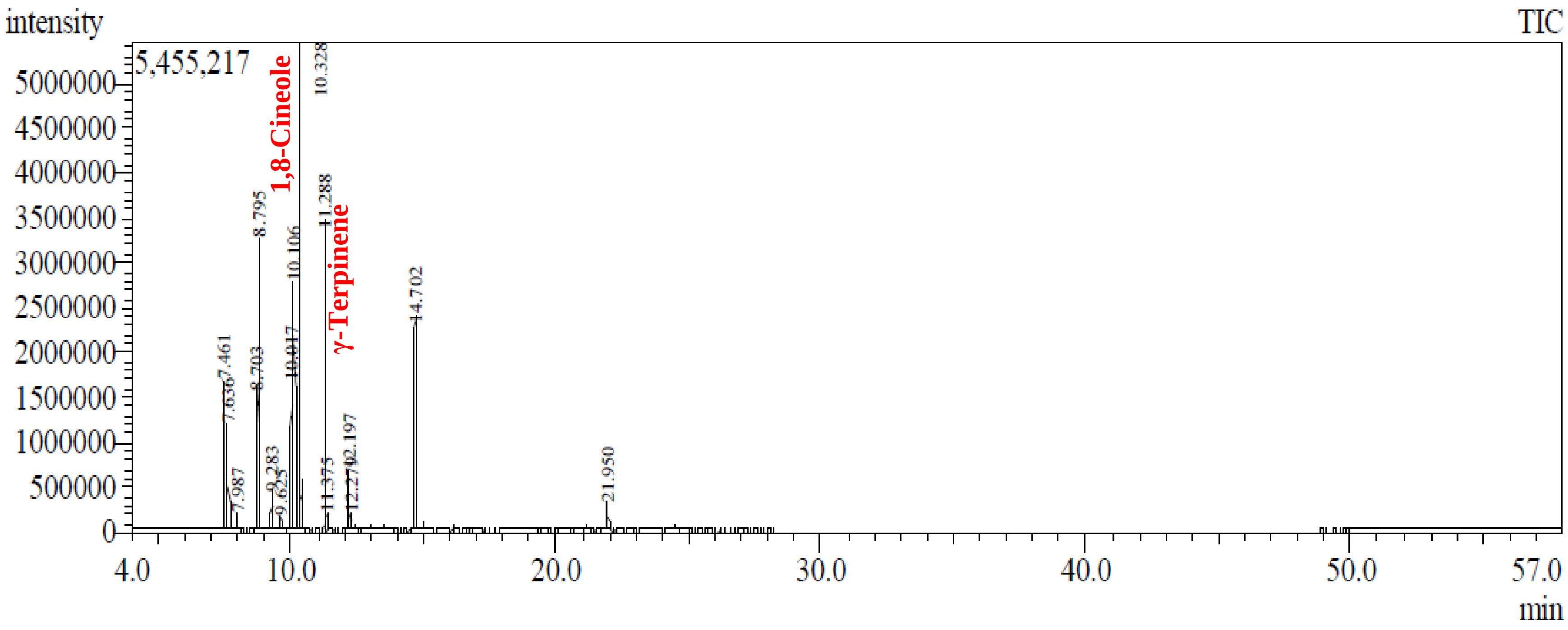

Figure S4: Total Ion Chromatogram of the Essential Oil Extracted from *Alpinia zerumbet* Rhizomes by Headspace

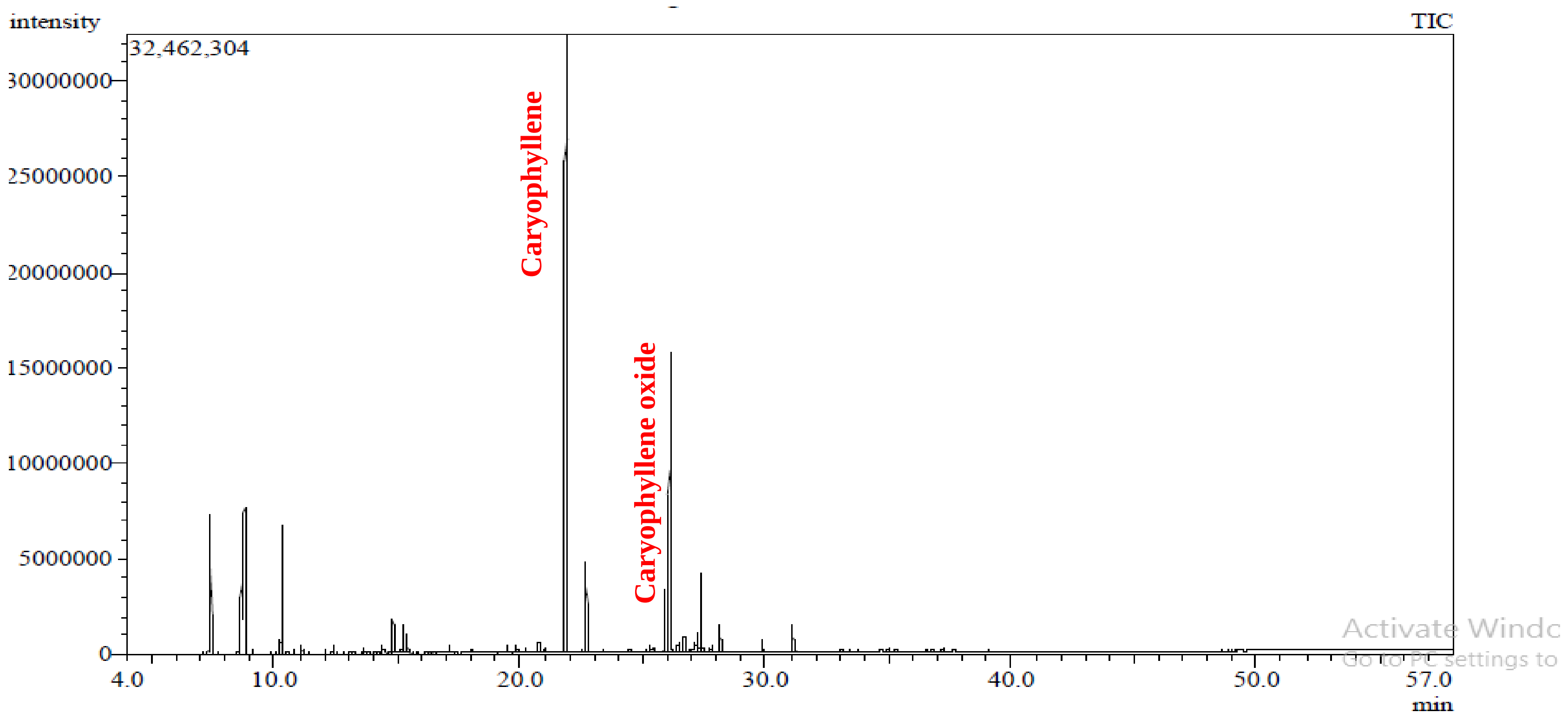

Figure S5: Total Ion Chromatogram of the Essential Oil Extracted from *Hedychium coronarium* Leaves by Hydrodistillation

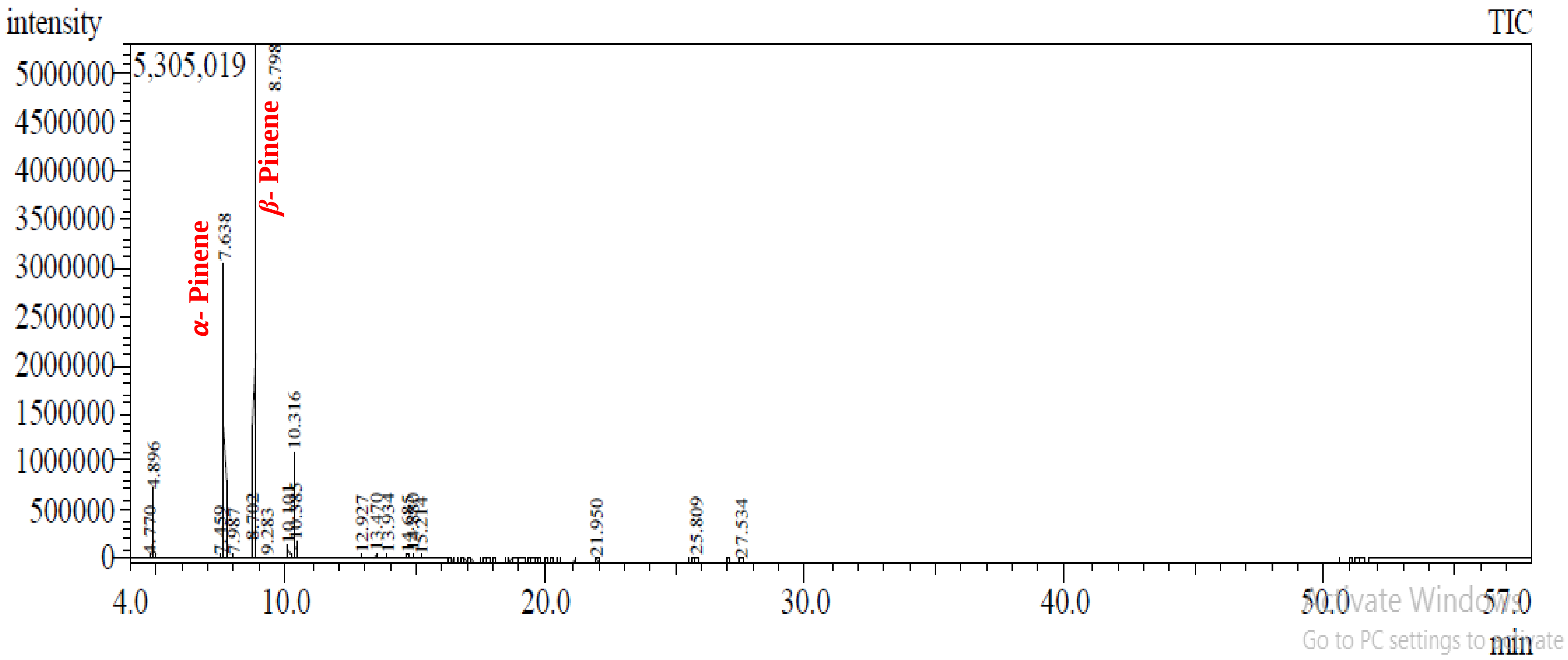

Figure S6: Total Ion Chromatogram of the Essential Oil Extracted from *Hedychium coronarium* Leaves by Headspace

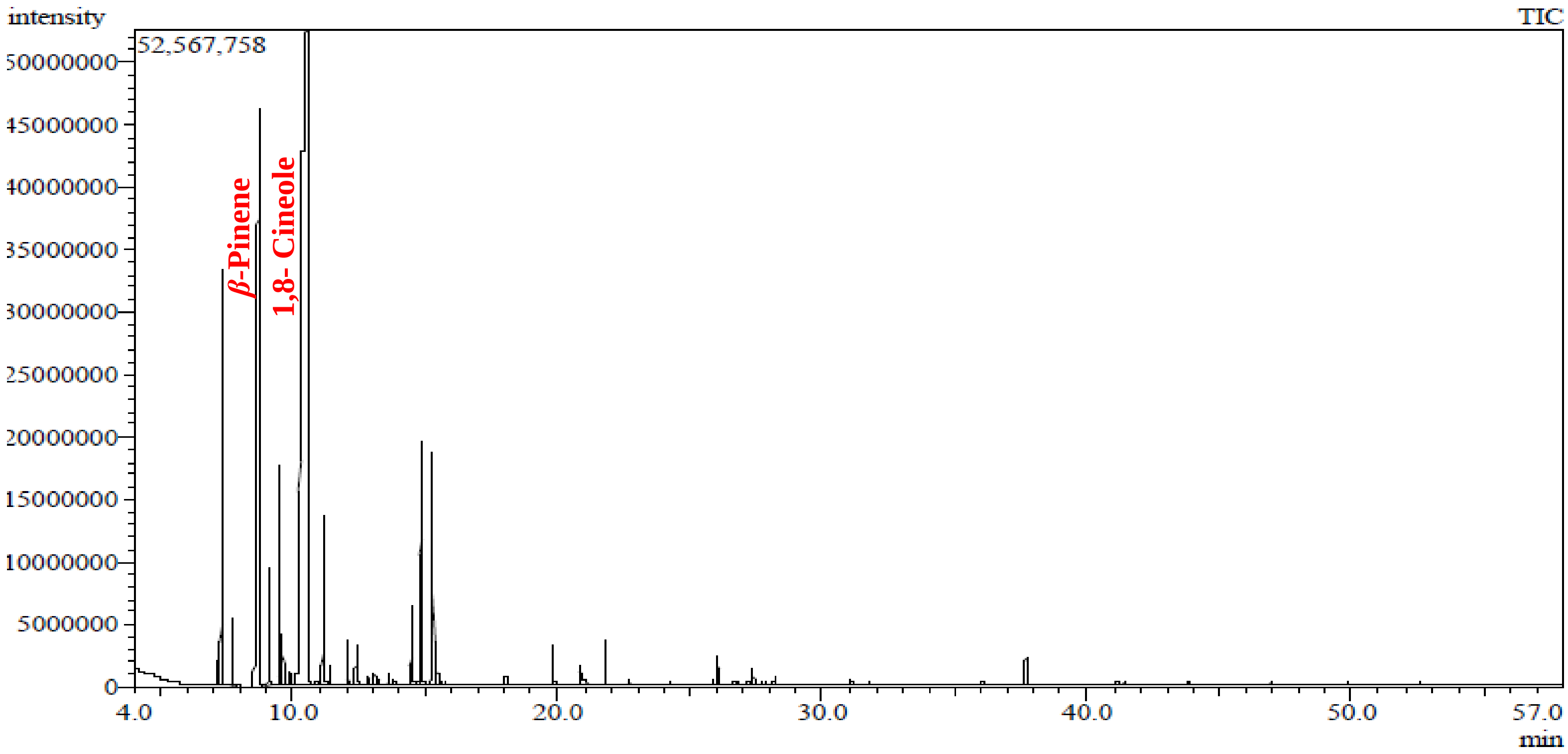

Figure S7: Total Ion Chromatogram of the Essential Oil Extracted from *Hedychium coronarium* Rhizomes by Hydrodistillation

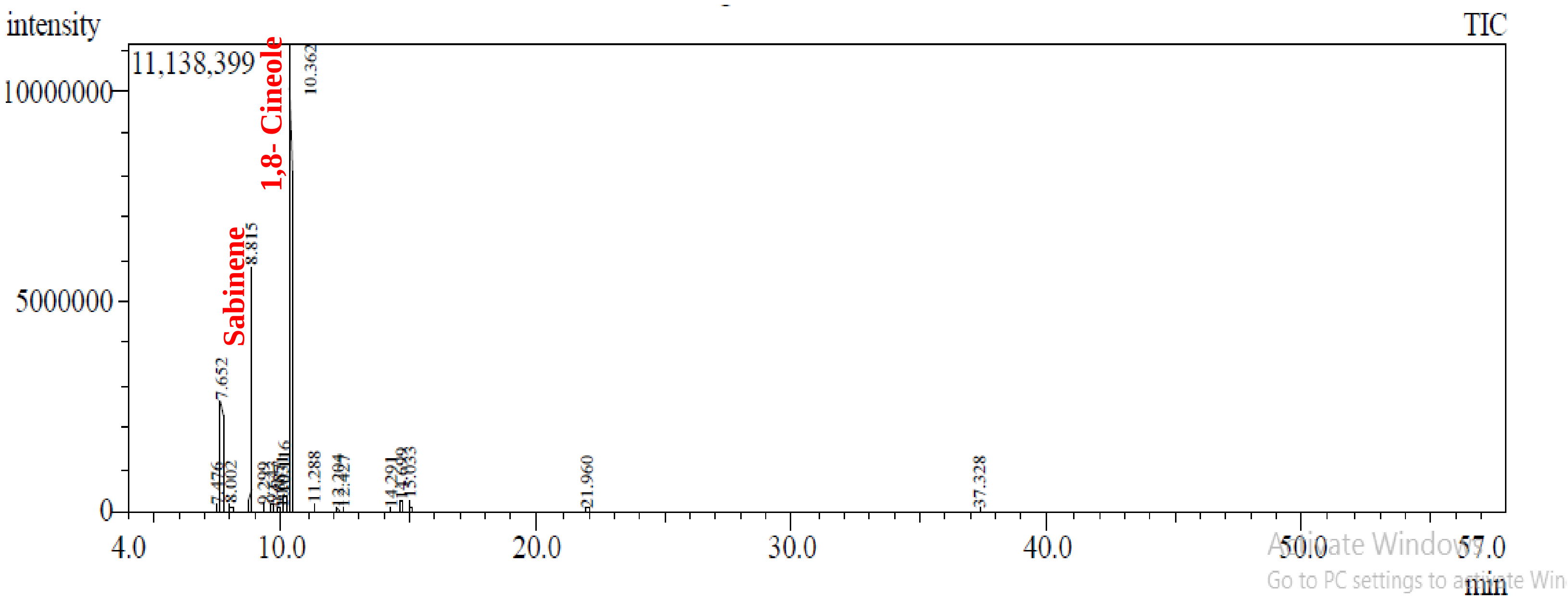

Figure S8: Total Ion Chromatogram of the Essential Oil Extracted from *Hedychium coronarium* Rhizomes by Headspace

(a)

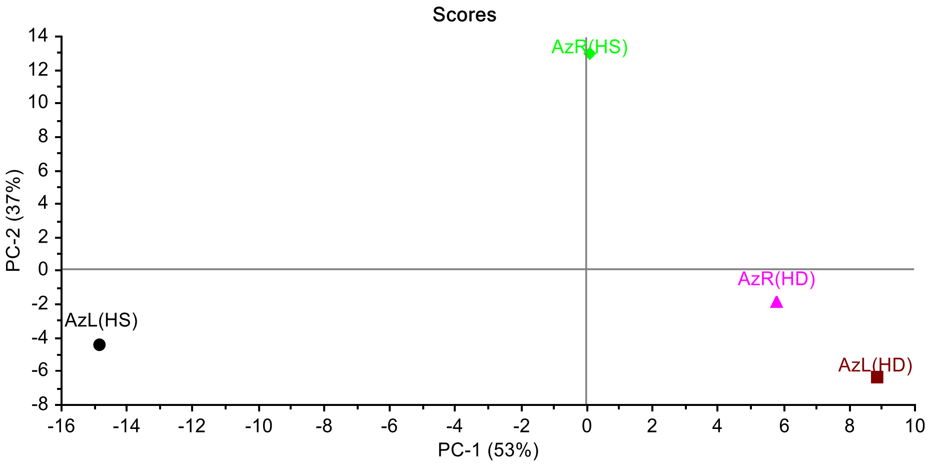

(b)

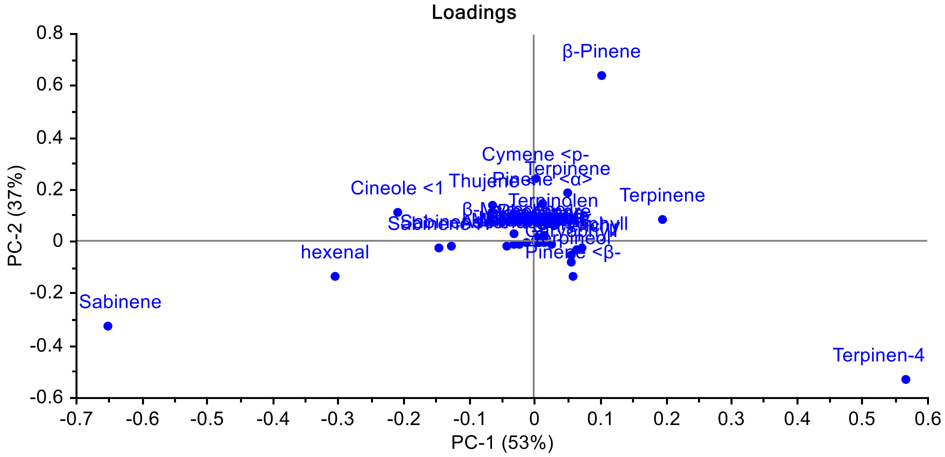

Complete linkage clustering using Squared Euclidean distance

(c)

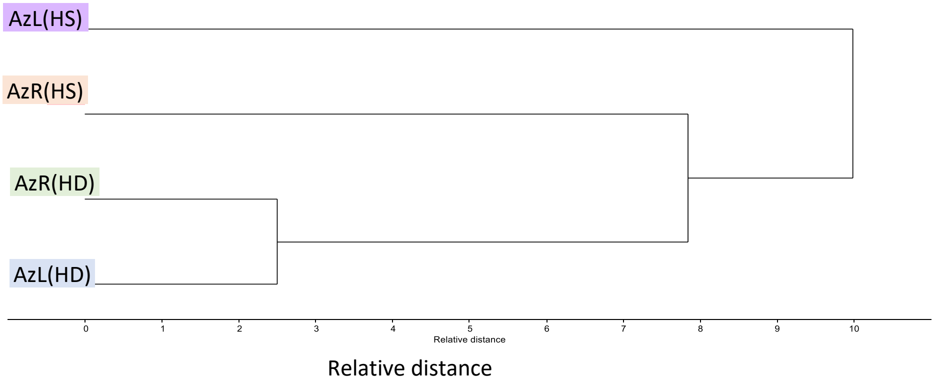

Figure S9: (a) PCA Score Plot, (b) PCA Loading Plot, (c) HCA Based on GC/MS Identification of the Chemical Compositions of the Essential Oils Extracted by Hydrodistillation and Headspace from Leaves and Rhizomes of *Alpinia zerumbet* Cultivated in Egypt

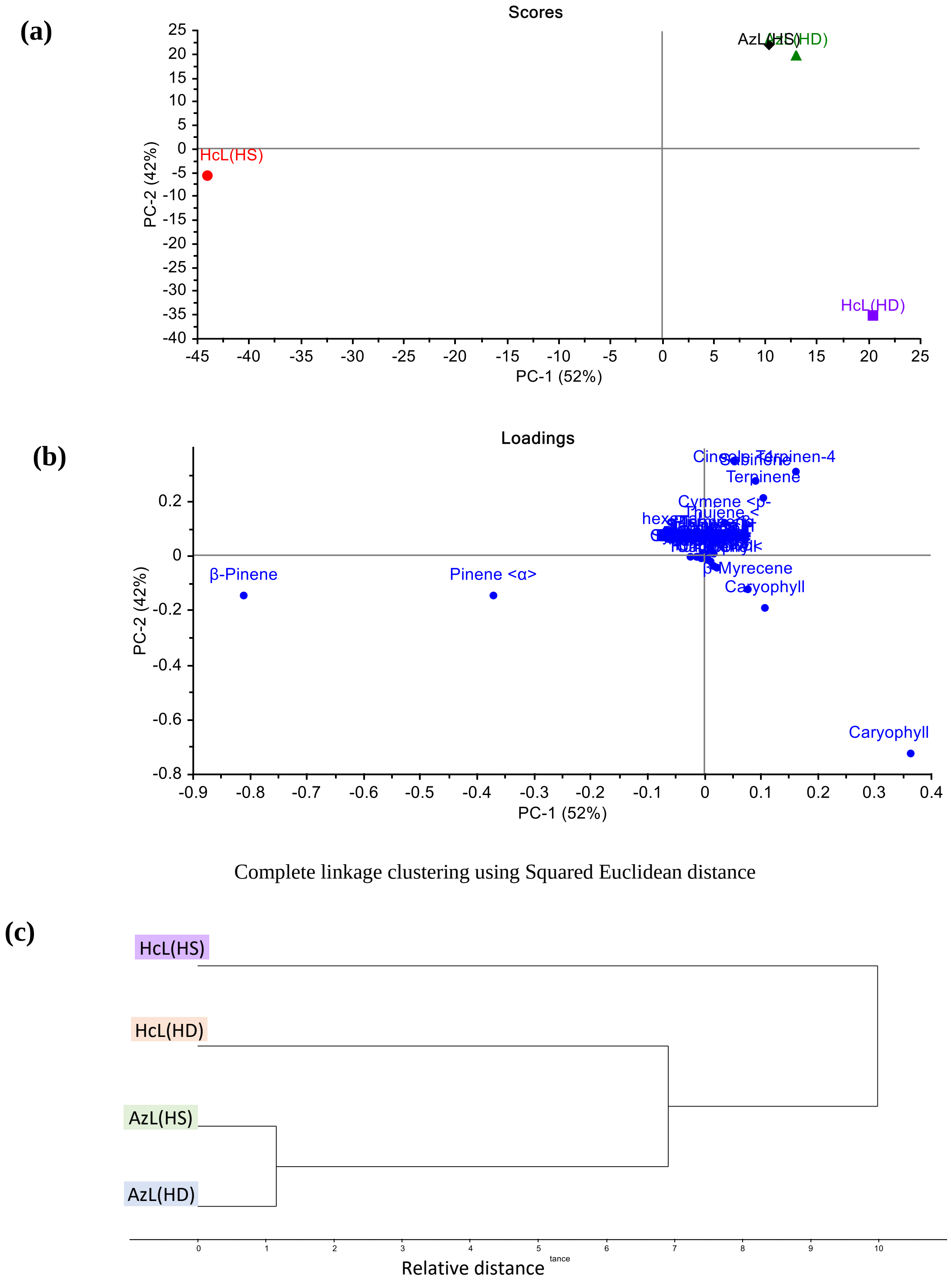

Figure S10: (a) PCA Score Plot, (b) PCA Loading Plot, (c) HCA Based on GC/MS identification of the Chemical Compositions of the Essential Oils Extracted by Hydrodistillation and Headspace from Leaves of *Hedychium coronarium* and *Alpinia zerumbet* Cultivated in Egypt

(a)

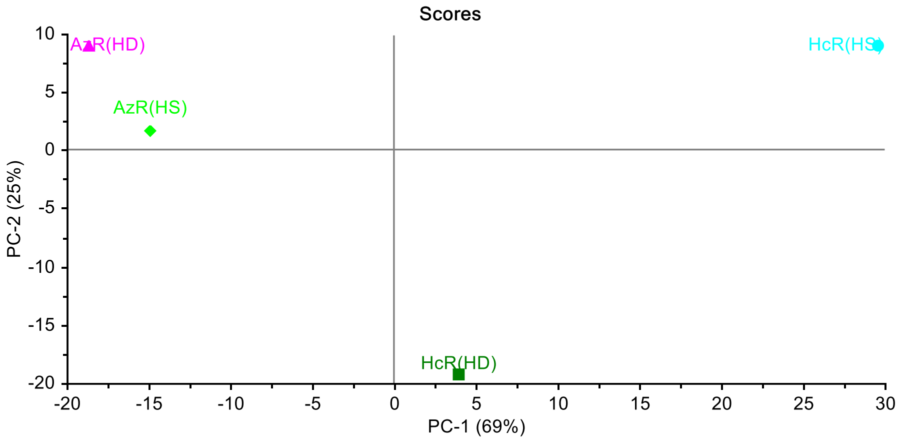

(b)

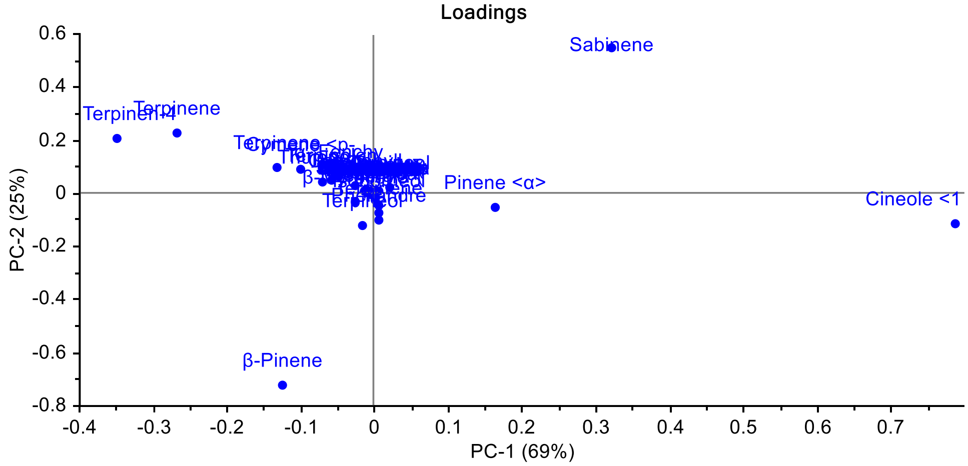

(c)

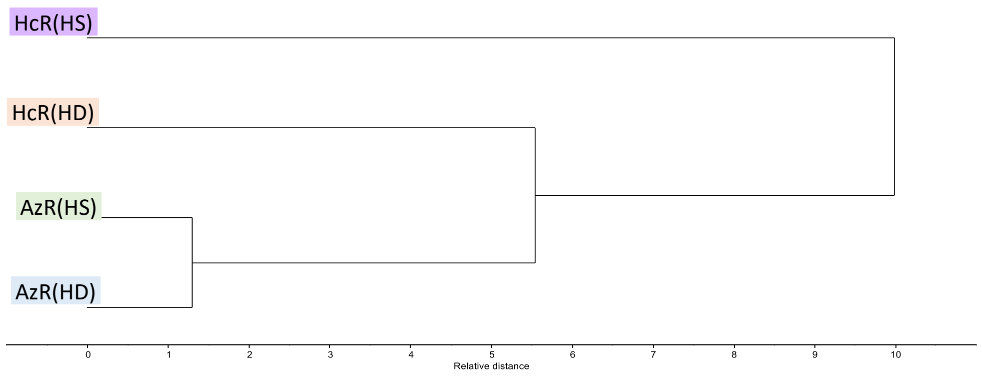

Figure 28: (a) PCA Score plot, (b) PCA Loading Plot, (c) HCA Based on GC/MS Identification of the Chemical Compositions of the Essential Oils Extracted by Hydrodistillation and Headspace from Rhizomes of *Hedychium coronarium* and *Alpinia zerumbet* Cultivated in Egypt
